# P-1353. Molecular Heterogeneity of *Stenotrophomonas maltophilia* Isolates Associated with Human Infections in the US and Colombia

**DOI:** 10.1093/ofid/ofae631.1530

**Published:** 2025-01-29

**Authors:** Maria F Mojica, David van Duin, Luis Felipe F Reyes, Lina Mendez, Gauri G Rao, Derrick Fouts, Robert Bonomo

**Affiliations:** Case Western Reserve University, Cleveland, Ohio; University of North Carolina at Chapel Hill, Chapel Hill, NC; Universidad de La Sabana, Chía, Cundinamarca, Colombia; Clinica Universidad de La Sabana, Chía, Cundinamarca, Colombia; USC Alfred E. Mann School of Pharmaceiti, Rancho Palos Verdes, California; J. Craig Venter Institute, Rockville, MD; Case Western, Cleveland, Ohio

## Abstract

**Background:**

*Stenotrophomonas maltophilia* (*Sma*) is an increasingly important opportunistic bacterial pathogen that commonly causes bloodstream infection (BSI) or pneumonia. *Sma* is intrinsically resistant to most antibiotics and may acquire additional resistance. Despite its significant clinical impact, *Sma* is remarkably understudied. Consequently, critical gaps persist in our understanding of *Sma*’s genomic and microbiological characteristics, which may impact treatment outcomes. Herein, we characterized 121 Sma isolates by whole genome sequencing (WGS).

**Figure 1.**
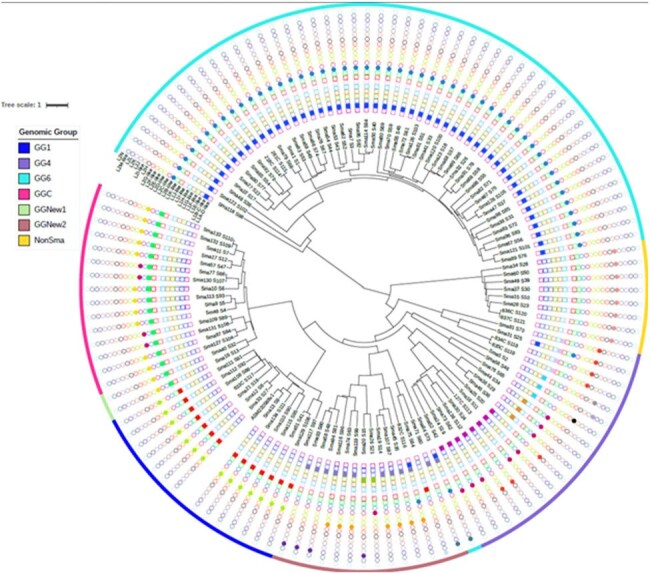
Putative association of specific types of L1 and L2 β-lactamases with genomic groups of Stenotrophomonas spp. A consensus UPGMA tree of core genomes shows the structure of the studied population (n = 121). Branch length represents the genetic distance, with the scale bar indicating the number of substitutions per nucleotide position. The genomic group designation is indicated by the colored internal ring. Presence/absence of L1 and L2 types is indicated by full or empty squares or circles, respectively.

**Methods:**

*Sma* isolates were subjected to WGS using Illumina technology. Genomes were annotated using Prodigal gene prediction software. Resistome was predicted using RGI, which uses the CARD database.

**Results:**

Phylogenetic analysis revealed that isolates were grouped into 8 genomic groups. Of those, GG6, GG4, GGC and GG1 represented 41%, 22%,18% and 17% isolates, respectively. Notably, we did not notice outstanding differences between isolates collected from different geographical locations or diverse biological sources, including from CF patients. Consistent with our previous report (PMID: 31266860), we observed a perfect correlation between GG6 and L1B-C-like and L2B-like B-lactamases (BLs). Further analysis demonstrated that these BLs display increased catalytic activities towards 3^rd^ gen. cephalosporins and carbapenems. Surprisingly, we also observed that all isolates from the GG1 harbored a specific type of L1 (L1J-like) and L2 (L2E), which are novel types of L1 and L2 BLs (Fig. 1). The most distant genomic group, presumably a new *Stenotrophomonas* species, clustered isolates which only harbored a new type of L2, L2I. The distribution of *qnr* and *mfpA* genes among the genomic groups of *Sma* does not seem to be segregated by genomic groups. However, an in-depth analysis of 11 selected GG6 (n = 6) and non-GG6 strains (n = 5) strains shows that the SmeS efflux pump and AAC(6')-Iz aminoglycoside modifying enzyme are exclusively associated with GG6.

**Conclusion:**

*Sma* strains belonging to the GG6, which has been exclusively associated with human infections and accounts for the majority of the isolates in the US, have a distinct resistome. The impact of *Sma* GGs with different resistomes on treatment outcomes is unknown.

**Disclosures:**

**David van Duin, MD, PhD**, Merck: Advisor/Consultant|Merck: Grant/Research Support|Pfizer: Advisor/Consultant|Qpex: Advisor/Consultant|Roche: Advisor/Consultant|Shionogi: Advisor/Consultant|Shionogi: Grant/Research Support **Gauri G. Rao, PharmD, MS**, Merck: Grant/Research Support **Robert Bonomo, MD**, Merck: Grant/Research Support|Shionogi: Grant/Research Support

